# Hepatocyte Growth Factor Activator: A Proteinase Linking Tissue Injury with Repair

**DOI:** 10.3390/ijms19113435

**Published:** 2018-11-01

**Authors:** Tsuyoshi Fukushima, Shuichiro Uchiyama, Hiroyuki Tanaka, Hiroaki Kataoka

**Affiliations:** 1 Section of Oncopathology and Regenerative Biology, Faculty of Medicine, University of Miyazaki, 5200 Kihara, Kiyotake 889-1692, Miyazaki, Japan; fukuchan@med.miyazaki-u.ac.jp (T.F.); hiroyuki_tanaka@med.miyazaki-u.ac.jp (H.T.); 2 Department of Surgery, Miyakonojyo Medical Association Hospital, 1364-1 Tarobocho, Miyakonojyo 885-0002, Miyazaki, Japan; s_uchiyama@med.miyazaki-u.ac.jp

**Keywords:** hepatocyte growth factor activator, HGF, MET, protease, tissue injury, tissue repair

## Abstract

Hepatocyte growth factor (HGF) promotes pleiotropic signaling through its specific receptor tyrosine kinase, MET. As such, it has important roles in the regeneration of injured tissues. Since HGF is produced mainly by mesenchymal cells and MET is expressed in most epithelial, endothelial and somatic stem cells, HGF functions as a typical paracrine growth factor. HGF is secreted as an inactive precursor (proHGF) and requires proteolytic activation to initiate HGF-induced MET signaling. HGF activator (HGFAC) is a serum activator of proHGF and produces robust HGF activities in injured tissues. HGFAC is a coagulation factor XII-like serine endopeptidase that circulates in the plasma as a zymogen (proHGFAC). Thrombin, kallikrein-related peptidase (KLK)-4 or KLK-5 efficiently activates proHGFAC. The activated HGFAC cleaves proHGF at Arg494-Val495, resulting in the formation of the active disulfide-linked heterodimer HGF. Macrophage stimulating protein, a ligand of RON, is also activated by HGFAC in vivo. Although HGFAC functions primarily at the site of damaged tissue, a recent report has suggested that activated HGFAC relays a signal to stem cells in non-injured tissues via proHGF activation in the stem cell niche. This review focuses on current knowledge regarding HGFAC-mediated proHGF activation and its roles in tissue regeneration and repair.

## 1. Activation of Hepatocyte Growth Factor (HGF) Precursor

HGF is a pleiotropic peptide growth factor that was discovered as a potent mitogen for cultured hepatocytes [1]. This molecule is identical to scatter factor, an independently identified molecule that dissociated epithelial cells and increased their migratory activity [2]. Now, it is well established that HGF plays a major role in the regeneration and repair of the liver as well as other organs, including the gastrointestinal tract, kidney, lung and nervous system [3,4,5,6]. HGF is a typical paracrine factor that is produced by mesenchymal cells in a precursor form (proHGF) that acts on MET-expressing parenchymal cells after its proteolytic activation [7]. In this context, regulation of the activating proteinase is a very important step in the control of HGF-MET signaling [8]. Indeed, the HGF molecule detected in normal tissues is primarily in a form of single-chain inactive proHGF [9,10].

Several proteinases have been reported to activate proHGF. All are serine proteinases and they can be categorized into two groups: (1) circulating proteinases that emerge after activation of the coagulation cascade (serum proteinases) and, (2) tissue proteinases that function as proHGF activators in localized pericellular microenvironments (cellular proteinases) (Table 1) [11]. ProHGF protein has a structure similar to plasminogen. Consequently, plasminogen activators, particularly urokinase-type plasminogen activator (uPA), were initially reported to be proHGF converting enzymes [12]. However, the processing by uPA was a stoichiometric reaction [13], not an enzymatic reaction, and thus, its efficacy to produce mature HGF was very low [13,14]. It is now believed that the major proHGF-converting proteinases are serum HGF activator (HGFAC) and cellular type II transmembrane serine proteinases (TTSP), particularly matriptase [11,14,15,16].

## 2. Discovery, Molecular Structure and Physiological Substrates of HGFAC

In the process of purifying recombinant human HGF from culture supernatants of Chinese hamster ovary cells transfected with the expression vector harboring full-length *HGF* cDNA, Shimomura et al. [17] noticed that fetal bovine serum (FBS) in the growth medium had a potent activity, converting the single-chain inactive proHGF to mature two-chain active HGF. In 1992, they purified the corresponding proteinase from FBS and named it HGFAC [17]. Shortly afterwards, the human counterpart of this novel proteinase was purified from human serum and its cDNA was cloned [18]. The cloning study revealed that HGFAC is a coagulation factor XII-like serine endopeptidase of the trypsin-like S1 family. Moreover, it was found to be initially synthesized as a single-chain inactive zymogen (proHGFAC) [18]. Indeed, the phylogeny of the cDNA sequences of *HGFAC* and factor XII (*F12*) genes indicated that the gene for factor XII evolved from duplication of the *HGFAC* gene [19,20]. It is noteworthy that proHGF protein is homologous to plasminogen and has the same ancestral gene as plasminogen [19]. Therefore, the HGFAC-HGF system likely evolved along with that of the coagulation and fibrinolysis systems, suggesting its primary role is involved in the host’s response to tissue injuries. In accordance with this assumption, thrombin efficiently converts proHGFAC to the active two-chain form of HGFAC [21].

ProHGFAC is primarily synthesized by hepatocytes and circulates in plasma [8]. It is a relatively abundant plasma protein with a mean concentration around 40 nM in healthy individuals. On the other hand, low but distinct expression of *HGFAC* mRNA has been reported in extrahepatic organs, including the gastrointestinal tract, kidney, lung and central nervous system [8]. Regarding the substrates for HGFAC, only two proteins have been reported in vivo: proHGF and pro-macrophage stimulating protein (proMSP) [8,22]. MSP is an HGF-like protein synthesized by the liver and circulating in plasma (2–5 nM) as an inactive precursor form (proMSP) [22,23,24]. HGFAC cleaves proMSP at the Arg483-Val484 bond and generates a two-chain active MSP that exerts its biological activity through RON receptor tyrosine kinase, expressed by macrophages, epithelial cells and cancer cells [23]. However, considering its relatively high concentration in the plasma, it would not be surprising if HGFAC had additional unknown physiological substrates in vivo. Further studies for the substrates and biological functions of HGFAC in vivo will be required.

## 3. Robust Activation of proHGF by HGFAC in Response to Tissue Injury and Inflammation

**Figure 1:**
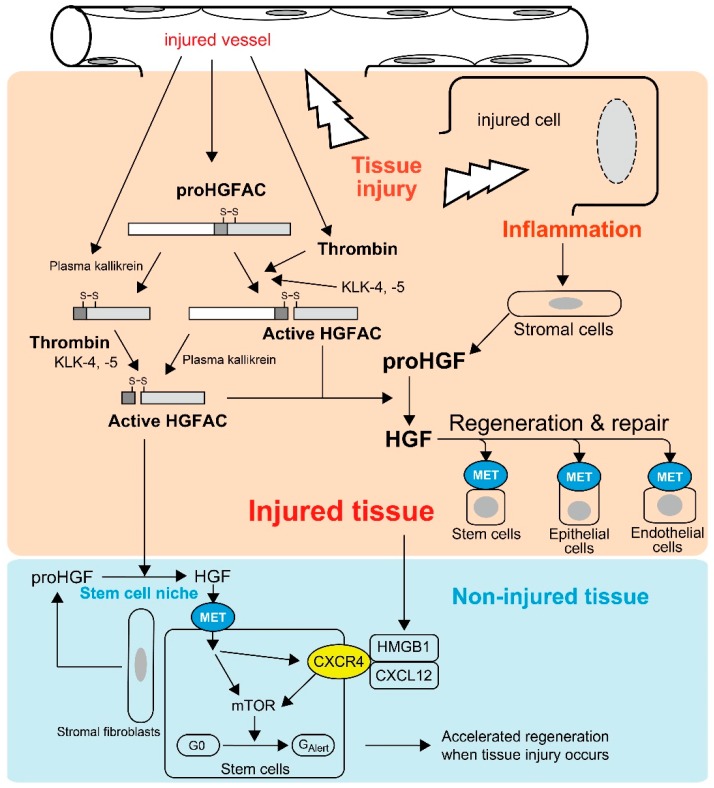
Tissue injury-induced activations of proHGF by HGFAC in response to tissue injury. Thrombin efficiently activates plasma-derived proHGFAC. KLK-4 and -5 also activate proHGFAC as well. Activated HGFAC is also released into the bloodstream and serves as an “alarmin” for tissue stem cells of non-injured tissue to prepare for the regeneration phase. Injured cell-derived HMGB1 also serves as a similar alarmin through CXCR4, and HGF-MET signaling upregulates CXCR4 expression in stem cells.

Most HGFAC proteins circulate in plasma as inactive zymogens [21]. HGFAC was initially discovered in bovine and human serum from which it was purified [8,17,18]. Supporting these observations, the serum activity of proHGF processing was severely attenuated in sera from *Hgfac* knockout mice, indicating that HGFAC is responsible for the proHGF-processing activity in serum [25]. The identification of a strong HGFAC activity in serum, but not plasma, implied that HGFAC-HGF-MET signaling is a system evoked by the activation of the coagulation cascade to promote the repair of injured tissue. This is also the case for HGFAC-MSP-RON signaling [8,11,21]. Indeed, proHGFAC is efficiently activated by thrombin, particularly in the presence of dextran sulfate, chondroitin sulfate and heparin [21] (Figure 1). These negatively-charged macromolecules are rich in the pericellular microenvironment as glycosaminoglycans. Of note, HGF is a heparin-binding growth factor and activated HGFAC acquires heparin affinity [18]. Thus, these proteins may co-localize with pericellular glycosaminoglycans, thereby efficiently forming localized machinery for the activation of HGF-MET signaling in the tissue microenvironment upon tissue injury (Figure 1).

The above hypothesis was confirmed by Miyazawa et al., who showed that robust activation of proHGF occurred exclusively at the site of tissue injury in their rat models of CCl_4_-induced liver injury and HgCl_2_-induced renal injury [9]. In these models, the processing of proHGF to active HGF was significantly suppressed by an anti-HGFAC neutralizing antibody [26]. Similarly, tissue injury induced robust activation of proHGF in acetic acid-induced and dextran sodium sulfate-induced mouse colitis models, and the activation was significantly attenuated in *Hgfac*-deleted mouse intestine accompanied with retarded repair and decreased survival of the mice [25]. HGFAC zymogen can also be activated efficiently by tissue proteases such as kallikrein-like peptidases (KLK), particularly KLK-4 or KLK-5 [27]. Furthermore, *Hgf* mRNA levels were increased in stromal cells by stimulation of inflammatory cytokines and growth factors, for example interleukin-1 (IL-1), tumor necrosis factor-α, platelet-derived growth factor and transforming growth factor-α [28]. Therefore, in addition to tissue injury-induced activation of HGFAC, post-injury inflammation would orchestrate the sustained activation of proHGF at the site of tissue injury (Figure 1).

**Figure 2:**
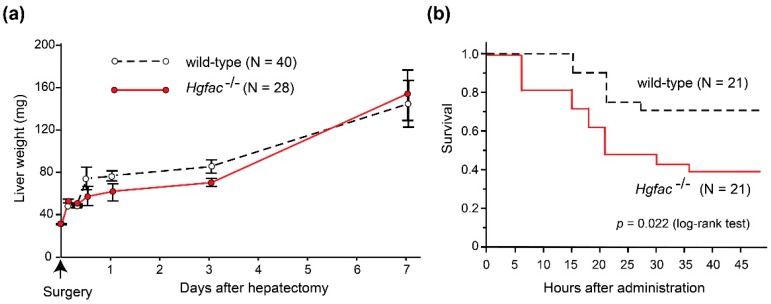
Effects of *Hgfac* deletion on liver regeneration and survival of C57BL6 mice after partial hepatectomy (**a**) and CCl_4_ treatment (**b**). (**a**) Effect of *Hgfac* deletion on liver weight gain after 70% partial hepatectomy. *N* = 40 for wild-type mice and 28 for *Hgfac*^−/−^ mice on the 7th day. No significant differences between two groups (two way repeated-measures analysis of variance). (**b**) Effect of *Hgfac* deletion on the survival of mice after CCl_4_ administration (2.5 μL/g, intraperitoneal injection). Kaplan-Meier survival curve is shown. *, *p* = 0.0216 (log-rank test); *N* = 21 for each group. All mice were maintained, treated, and sacrificed in accordance with the protocols and regulations of Miyazaki University Institutional Animal Care and Use Committee.

Experimental evidence supports the significance of inflammation in sustaining HGF activities. In rat liver injury models, CCl_4_ treatment induced significant activation of proHGF in the liver tissue [9]. However, partial hepatectomy did not induce robust proHGF activation [29]. In accordance with those findings, we observed that liver regeneration after CCl_4_-induced damage was delayed in *Hgfac* knockout mice, whereas that after partial hepatectomy was not affected by the deletion of *Hgfac* (Figure 2). Taken altogether, we propose that HGFAC-dependent sustained HGF-MET signaling is a system evoked by tissue damage and orchestrated by the subsequent inflammation to accelerate the repair process. In contrast, HGFAC is not essential for normal development or tissue homeostasis, so that *Hgfac* knockout mice showed no developmental defects and matured normally and were fertile [25].

## 4. Emerging Alarmin Function of HGFA upon Tissue Injury

Recently, Rodgers et al. observed activated circulating HGFAC in plasma after experimental tissue injury in mice [30]. They revealed that circulating HGFAC relays a signal to quiescent muscle stem cells and fibro-adipogenic progenitors in non-injured tissues to stimulate their transition from the G_0_ state to a primed G_Alert_ state, which is mediated by HGF-MET-mTORC1 signaling (Figure 1) [30,31]. Therefore, HGFAC likely stimulates the G_0_ → G_Alert_ transition of stem cells through activation of pericellular proHGF in the stem cell niche. Other stem cells such as epidermal, mesenchymal, hepatic and hematopoietic stem cells also express MET [5,6,30,32], which implies these stem cells may also respond to HGFAC in a similar fashion. Therefore, the function of HGFAC in tissue repair may not be restricted to the site of injury and HGFAC may function as a kind of alarmin. Alarmins are endogenous molecules released upon tissue damage to activate host responses, particularly inflammatory responses [33,34]. Upon tissue injury, activated HGFAC is systematically released and likely functions as a new type of alarmin to prepare for the regeneration phase (Figure 1). Very recently, high mobility group box 1 (HMGB1) complexed with CXCL12 was identified as another alarmin to transition stem cells from G_0_ to G_Alert_ through CXCR4 [35]. Interestingly, inhibition of MET activity reduced the expression of surface CXCR4 in murine muscle stem cells, suggesting that the HGFAC-HGF-MET pathway and the HMGB1-CXCL12-CXCR4 pathway may be complementary for accelerated tissue regeneration (Figure 1) [35].

## 5. Regulation of HGFAC Activity by Endogenous Proteinase Inhibitors

**Figure 3:**
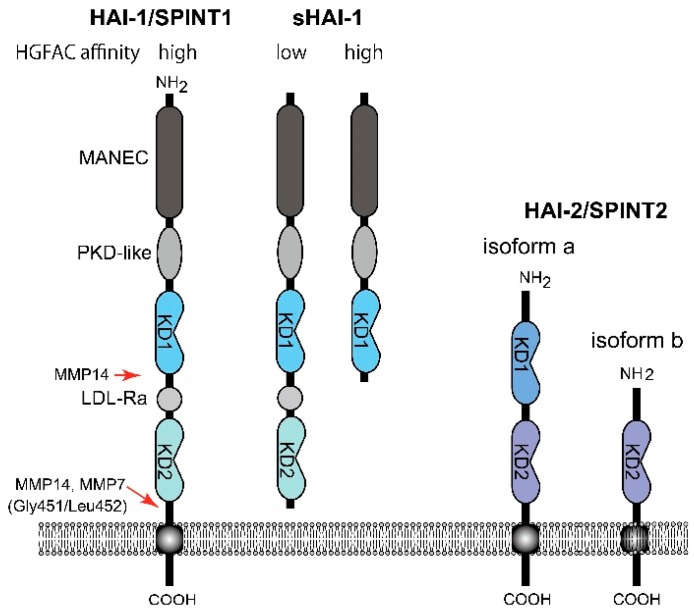
Molecular structure of HAI-1/SPINT1 and HAI-2/SPINT2. Whereas the membrane-form HAI-1/SPINT1 and sHAI-1 only with KD1 show high affinity to HGFAC, sHAI-1 having both KD1 and KD2, shows decreased affinity to HGFAC. MANEC, N-terminus with eight-cysteines domain; PKD-like, polycystic kidney disease domain-like domain; LDL-Ra, low density lipoprotein receptor class A domain; KD, Kunitz domain; MMP, matrix metalloprotease.

To date, two endogenous proteinase inhibitors have been shown to regulate HGFAC activity in a cell-based assay and in vivo. One is protein C inhibitor (PCI, also known as SERPINA5), a circulating serpin-type inhibitor produced by the liver and also locally expressed by reproductive organs [36]. The other one is a Kunitz-type type 1 transmembrane inhibitor, HGFAC inhibitor type 1 (HAI-1)/serine peptidase inhibitor, Kunitz type 1 (SPINT1). It is widely expressed by epithelial cells and regulates HGFAC activity in the pericellular microenvironment [16,37,38]. HAI-1/SPINT1 has two extracellular Kunitz-type serine proteinase inhibitor domains: an N-terminal Kunitz domain (KD1) and a C-terminal Kunitz domain (KD2) (Figure 3). HAI-2/SPINT2 is another type 1 transmembrane Kunitz-type inhibitor with Kunitz domains homologous to those of HAI-1/SPINT1. Although HAI-2/SPINT2 was also identified as a protein efficiently inhibiting HGFAC [39], the physiological role of HAI-2/SPINT2 in the regulation of HGFAC is still a matter of debate [16]. Evidence indicates that only HAI-1/SPINT1 can complex with activated HGFAC on the surface of epithelial cells that are expressing both HAIs, suggesting that HAI-2/SPINT2 does not function as an HGFAC inhibitor on the cell surface [40]. Furthermore, HAI-2/SPINT2 immunoreactivity is exclusively observed in the cytoplasm [16,41,42], likely residing in the endoplasmic reticulum [43]. Those findings suggest a primary role of HAI-2/SPINT2 for regulation of serine proteinase activities in the endoplasmic reticulum and intracellular secretory pathways. Thus, HAI-2/SPINT2 may not be a physiological inhibitor of serum HGFAC. On the other hand, HAI-2/SPINT2 may function as an HGFAC inhibitor to suppress HGF-MET signaling in cancer tissues [44]. Further studies are required to define the roles of HAI-2/SPINT2 in HGF-MET signaling in vivo.

Thrombin catalyzes proHGFAC in the presence of heparin, which appears to be inhibited by PCI. More importantly, PCI directly inhibits HGFAC in the absence of heparin [36]. The in vivo role of PCI in the regulation of HGFAC activity was further demonstrated in a partial hepatectomy model using human PCI-transgenic mice that mimic PCI expression in humans [45]. The PCI-HGFAC complex was detectable in human plasma and the concentration of the complex was significantly higher in hepatocellular carcinoma patients (60 ± 20 pM) than in normal individuals (27 ± 10 pM) [36]. Therefore, PCI serves as a major circulating inhibitor of HGFAC.

On the other hand, HAI-1/SPINT1 is a cell surface inhibitor of HGFAC that regulates pericellular HGFAC activity [16,40]. It also regulates cell surface activators of proHGF, such as matriptase and hepsin [14,16]. In contrast to PCI that forms an irreversible complex with HGFAC, the inhibition of HGFAC by HAI-1/SPINT1 is mediated by a reversible complex formation of KD1 with the catalytic site of active HGFAC [40]. It is noteworthy that the cell surface HAI-1/SPINT1-HGFAC complex can be released by the cleavage of HAI-1/SPINT1 between KD2 and the transmembrane domain, followed by recovery of substantial HGFAC activity (around 70% of the initial activity) in the pericellular space [40]. This phenomenon is caused by markedly decreased affinity of the shed form HAI-1/SPINT1 (sHAI-1) compared to that of the membrane-anchored form to active HGFAC (Figure 3) [46]. This observation implies paradoxical functions of HAI-1/SPINT1 in regard to pericellular HGFAC activity. HAI-1/SPINT1 is a cell surface inhibitor of HGFAC, but may serve as a reservoir of active HGFAC supplying pericellular HGFAC activity if the ectodomain shedding of HAI-1/SPINT1 is increased. This shedding is metalloproteinase-dependent and accelerated by IL-1 stimulation [40]. Membrane-type 1 matrix metalloproteinase (MT1-MMP, also known as MMP14) and MMP-7 reportedly cleave Gly451/Leu452 of HAI-1/SPINT1 to release the low affinity form sHAI-1 having both KD1 and KD2 [47,48]. ADAMs (a disintegrin and metalloprotease) may also be involved in shedding, which should be clarified in future studies. It should be noted that a short form of sHAI-1 that has only KD1 efficiently inhibits HGFAC, indicating that KD1 is the functional domain for the inhibition of HGFAC; KD2 with a free N-terminus interferes with the binding of KD1 to HGFAC [46,49,50].

## 6. Impaired HGFAC Function and Diseases

Active HGF is an important effector of cellular survival and migration as well as anti-fibrotic actions [3,4,5]. Consequently, impaired proHGF activation in damaged tissue may significantly disturb subsequent tissue repair. For example, *Hgfac* knockout mice are more susceptible to acetic acid-induced or dextran sulfate sodium-induced acute colitis compared to wild-type mice, and initial restitution by epithelial cells on the ulcer surface was significantly delayed in the mutant mice [25]. This restitution step likely requires HGF activity (through MET signaling) to potently stimulate proliferation and migration of gastrointestinal epithelial cells [4]. In fact, epithelial cells undergoing restitution on gastrointestinal ulcers showed enhanced MET phosphorylation in human tissue sections [51]. In accordance with these observations, the relationship between a single nucleotide polymorphism (SNP) in the *HGFAC* gene and gastrointestinal mucosal disorders has been suggested in humans. A reverse genetic study using electronic medical records indicated a positive relationship between missense SNP in the *HGFAC* gene (rs16844401; Arg516Gln in HGFAC isoform 1 that is identical to Arg509Gln in isoform 2 [11]) and a clinical diagnosis related to gastrointestinal bleeding [52]. In addition, a genome-wide screening study indicated another SNP (rs2073505; synonymous codon) may increase susceptibility for inflammatory bowel disease [53].

Experimental evidence suggests that an imbalance between HGFAC and HAIs may be involved in idiopathic pulmonary fibrosis (IPF) [54]. Lung fibroblasts normally express proHGF, HGFAC, HAI-1/SPINT1 and HAI-2/SPINT2. In contrast, those from IPF patients have a reduced capacity to activate proHGF in vitro, a consequence of a low expression level of HGFAC and high levels of HAI-1/SPINT1 and HAI-2/SPINT2 [55]. Similar imbalances were also suggested in a mouse bleomycin-induced lung injury model, in which lung *Spint1* mRNA expression and HAI-1/SPINT1 content in the bronchoalveolar lavage fluid were markedly increased compared to control mice. Thus, during fibrosis, an imbalance between HGFAC and HAI-1 became apparent, accompanying decreased proHGF activation [56]. In a study of chronic skin wounds, the beneficial effects of increased HGF and HGFAC expression could be blocked by increased expression of HAI-1/SPINT1 [57]. In addition, enhanced immunoreactivity of HAI-1/SPINT1 occurs in the gastrointestinal epithelium at the edge of ulcers [40], periportal hepatocytes of advanced primary biliary cholangitis (PBC), fulminant hepatitis [38] and also in the interlobular biliary epithelium of PBC patients [58]. However, it remains to be clarified whether augmented HAI-1/SPINT1 expression in fact suppresses the HGFAC-mediated proHGF activation in these cells. Another interesting relationship was observed between HGFAC expression and epilepsy. Decreased cortical *HGFAC* mRNA levels were correlated with increased frequency of temporal lobe epilepsy [59].

On the other hand, excess and persistent HGFAC activity likely occurs in tumor tissues, as tissue destruction, inflammation and enhanced procoagulant activity are unavoidable in solid tumors [60,61,62]. Considering the important roles of HGF-MET signaling in invasive growth, drug resistance and maintaining the stem cell phenotype of tumor cells, targeting proHGF activation may provide additional options to overcome the malignant phenotypes of tumor cells. The significance of HGFAC in tumor tissues has been reviewed elsewhere [15,61]. Although cell surface proHGF-activating TTSPs are primary targets to suppress excess proHGF activation in solid tumors [63], additional targeting of HGFAC would also be critically required [11,16,63].

## 7. Conclusions and Future Perspectives

There exist redundant mechanisms for activation of proHGF in vivo, including serum serine proteinases such as HGFAC, factor XIIa and XIa, as well as cellular TTSPs, such as matriptase and hepsin [16]. However, as summarized above, HGFAC is a primary activator of proHGF at the site of tissue injury, promoting accelerated healing of the damaged tissue. Moreover, HGFAC generated in injured tissues may function as alarmin that relays a signal to quiescent tissue stem cells distal to the injured site to stimulate their transition into a primed G_Alert_ state [30]. Therefore, pretreatment with recombinant HGFAC may be a promising option to accelerate the repair process after a surgical procedure. Furthermore, recombinant HGFAC may also have relevance in therapies of patients with refractory wounds or persistent inflammation. 

In summary, HGFAC is a proteinase that links tissue injury with subsequent regeneration and repair of tissues. The information described in this review provides opportunities to develop novel targeted approaches to accelerate tissue repair and treatment of refractory wounds.

## Figures and Tables

**Table 1:** Major proHGF-activating proteinases [11,14,16].

Proteinase	Localization	RA	RA+	Producing Organ/Tissue
**HGFAC**	blood	1.0	5.0	Liver, (brain, GI tract, etc.)
**Factor XIIa**	blood	0.02	0.7	Liver
**Factor XIa**	blood	0.015	NA	Liver
**Matriptase**	cell surface	2.07	NA	Epithelial tissues
**Hepsin**	cell surface	0.074	NA	Liver, Kidney, Inner ear
**HAT**	cell surface	0.02–0.06	NA	Respiratory epithelium

RA, relative activity; RA+, relative activity in the presence of high molecular weight dextran sulfate; GI tract, gastrointestinal tract; HAT, human airway trypsin-like protease; NA, not applicable.

## Abbreviations

HGF	Hepatocyte growth factor
HGFAC	Hepatocyte growth factor activator
KLK	Kallikrein-related peptidase
uPA	Urokinase-type plasminogen activator
HAT	Human airway trypsin-like protease
FBS	Fetal bovine serum
MSP	Macrophage stimulating protein
HMGB1	High mobility group box 1
PCI	Protein C inhibitor
HAI-1	Hepatocyte growth factor activator inhibitor type 1
SPINT1	Serine peptidase inhibitor, Kunitz type 1
HAI-2	Hepatocyte growth factor activator inhibitor type 2
SPINT2	Serine peptidase inhibitor, Kunitz type 2
KD	Kunitz domain
MANEC	N-terminus with eight-cysteines
PKD	Polycystic kidney disease
LDL-Ra	Low density lipoprotein receptor class A
MMP	Matrix metalloprotease
MT1-MMP	Membrane type-1 matrix metalloprotease
